# Rotating Night Shift Work and Risk of Type 2 Diabetes: Two Prospective Cohort Studies in Women

**DOI:** 10.1371/journal.pmed.1001141

**Published:** 2011-12-06

**Authors:** An Pan, Eva S. Schernhammer, Qi Sun, Frank B. Hu

**Affiliations:** 1 Department of Nutrition, Harvard School of Public Health, Boston, Massachusetts, United States of America; 2 Department of Epidemiology, Harvard School of Public Health, Boston, Massachusetts, United States of America; 3 Channing Laboratory, Department of Medicine, Brigham and Women's Hospital and Harvard Medical School, Boston, Massachusetts, United States of America; Lund University Diabetes Centre, Sweden

## Abstract

**Background:**

Rotating night shift work disrupts circadian rhythms and has been associated with obesity, metabolic syndrome, and glucose dysregulation. However, its association with type 2 diabetes remains unclear. Therefore, we aimed to evaluate this association in two cohorts of US women.

**Methods and Findings:**

We followed 69,269 women aged 42–67 in Nurses' Health Study I (NHS I, 1988–2008), and 107,915 women aged 25–42 in NHS II (1989–2007) without diabetes, cardiovascular disease, and cancer at baseline. Participants were asked how long they had worked rotating night shifts (defined as at least three nights/month in addition to days and evenings in that month) at baseline. This information was updated every 2–4 years in NHS II. Self-reported type 2 diabetes was confirmed by a validated supplementary questionnaire. We documented 6,165 (NHS I) and 3,961 (NHS II) incident type 2 diabetes cases during the 18–20 years of follow-up. In the Cox proportional models adjusted for diabetes risk factors, duration of shift work was monotonically associated with an increased risk of type 2 diabetes in both cohorts. Compared with women who reported no shift work, the pooled hazard ratios (95% confidence intervals) for participants with 1–2, 3–9, 10–19, and ≥20 years of shift work were 1.05 (1.00–1.11), 1.20 (1.14–1.26), 1.40 (1.30–1.51), and 1.58 (1.43–1.74, *p-*value for trend <0.001), respectively. Further adjustment for updated body mass index attenuated the association, and the pooled hazard ratios were 1.03 (0.98–1.08), 1.06 (1.01–1.11), 1.10 (1.02–1.18), and 1.24 (1.13–1.37, *p*-value for trend <0.001).

**Conclusions:**

Our results suggest that an extended period of rotating night shift work is associated with a modestly increased risk of type 2 diabetes in women, which appears to be partly mediated through body weight. Proper screening and intervention strategies in rotating night shift workers are needed for prevention of diabetes.

*Please see later in the article for the Editors' Summary*

## Abstract 2

An Pan and colleagues examined data from two Nurses' Health Studies and found that extended periods of rotating night shift work were associated with a modestly increased risk of type 2 diabetes, partly mediated through body weight.

## Editors' Summary

**Background:**

Around 346 million people worldwide have diabetes—a chronic disease affecting blood glucose levels, which over time may lead to serious damage in many body systems. In 2004, an estimated 3.4 million people died from consequences of high blood sugar, with more than 80% of deaths occurring in low-and middle-income countries. Type 2 diabetes accounts for 90% of people with diabetes and is largely the result of excess body weight and physical inactivity, which causes the body to use insulin ineffectively. One strategy in the public health response to the increasing prevalence and incidence of type 2 diabetes is to focus on the prevention and management of obesity by targeting risk factors of obesity.

Previous studies have suggested that rotating night shift work, which is common and becoming increasingly prevalent in countries worldwide, is associated with an increased risk of obesity and metabolic syndrome, conditions closely related to type 2 diabetes.

**Why Was This Study Done?:**

Some studies have investigated the association between rotating night shift work and type 2 diabetes but have experienced methodological problems (such as minimal information on the rotating shift work, small sample sizes, and limited study populations), which make interpretation of the results difficult. In this study, the researchers attempted to overcome these methodological issues by prospectively examining the relationship between duration of rotating night shift work and risk of incident type 2 diabetes and, also if the duration of shift work was associated with greater weight gain, in two large cohorts of women in the United States.

**What Did the Researchers Do and Find?:**

The researchers used data from the Nurses' Health Study I (NHS I, established in 1976 and included 121,704 women) and the Nurses' Health Study II (NHS II, established in 1989 and included 116,677 women), in which participating women completed regular questionnaires about their lifestyle practices and the development of chronic diseases. In both studies, the women also gave information about how long they had done rotating night shifts work (defined as at least three nights/month in addition to 19 days and evenings in that month), and this information was updated at regular intervals over the study follow-up period (18 years). The comparison group was women who did not report a history of rotating night shift work.

To assess the incidence of diabetes in both cohorts, the researchers sent a supplementary questionnaire to women who reported a diagnosis of diabetes, which asked about the symptoms, diagnostic tests, and medical management: if at least one of the National Diabetes Data Group criteria was reported, the researchers considered confirmed a diagnosis of type 2 diabetes. The researchers then used statistical methods (time-dependent Cox proportional hazards models) to estimate the hazard ratios of the chance of women working rotating shifts developing type 2 diabetes as a ratio of the chance of women not working rotating shifts developing diabetes.

The researchers found that in NHS I, 6,165 women developed type 2 diabetes and in NHS II 3,961 women developed type 2 diabetes. Using their statistical models, the researchers found that the duration of rotating night shift work was strongly associated with an increased risk of type 2 diabetes in both cohorts. The researchers found that in both cohorts, compared with women who reported no rotating night shift work, the HR of women developing type 2 diabetes, increased with the numbers of years working rotating shifts (the HRs of working rotating shifts for 1–2, 3–9, 10–19, and ≥20 years were 0.99, 1.17, 1.42, and 1.64, respectively, in NHS I, and in NHS II, 1.13, 1.34, 1.76, and 2.50, respectively). However, these associations were slightly weaker after the authors took other factors into consideration, except for body mass index (BMI).

**What Do These Findings Mean?:**

These findings show that in these women, there is a positive association between rotating night shift work and the risk of developing type 2 diabetes. Furthermore, long duration of shift work may also be associated with greater weight gain. Although these findings need to be confirmed in men and other ethnic groups, because a large proportion of the working population is involved in some kind of permanent night and rotating night shift work, these findings are of potential public health significance. Additional preventative strategies in rotating night shift workers should therefore be considered.

**Additional Information:**

Please access these Web sites via the online version of this summary at http://dx.doi.org/10.1371/journal.pmed.1001141.

- This study is further discussed in a *PLoS Medicine* Perspective by Mika Kivimki and colleagues
- Wikipedia has information about the Nurses’ Health study (note that Wikipedia is a free online encyclopedia that anyone can edit; available in several languages)
- Detailed information about the Nurses’ Health Study is available
- The World Health Organization provides comprehensive information about all kinds of diabetes
- For more information about diabetes that is useful for patients see Diabetes UK

## Introduction

Rotating night shift work is common and is becoming increasingly prevalent in industrialized nations [1]. Several studies have suggested that rotating night shift work is associated with an increased risk of obesity [2] and metabolic syndrome [3],[4], conditions closely related to type 2 diabetes. However, few studies have investigated the association between rotating night shift work and type 2 diabetes. Mikuni et al. [5] reported a higher prevalence of diabetes among rotating night shift workers in a male Japanese population. Several cohort studies reported an increased risk of impaired glucose metabolism [3],[6], and two prospective studies in male Japanese workers have revealed that alternation/shift worker had an increased risk of impaired glucose metabolism and diabetes compared with day workers [7],[8]. However, information on the duration of rotating night shift work was not available in these studies, the sample sizes were relatively small, and the study population was limited to Japanese males.

Therefore, we prospectively examined the relationship between duration of rotating night shift work and risk of incident type 2 diabetes in two large cohorts of women in the United States: the Nurses' Health Study (NHS) I and NHS II, with 18–20 y of follow-up. In a secondary analysis, we examined whether duration of shift work was associated with greater weight gain.

## Methods

### Ethics Statement

The study protocol was approved by the institutional review boards of Brigham and Women's Hospital and Harvard School of Public Health (Boston, Massachusetts, United States).

### Study Population

We used data from two prospective cohort studies: NHS I (established in 1976, *n = *121,704) [9], and NHS II (established in 1989, *n = *116,677) [10]. In both cohorts, questionnaires were administered at baseline and biennially thereafter, to collect and update information on lifestyle practice and occurrence of chronic diseases. The follow-up rates in these cohorts have both exceeded 90%. Women who answered the 1988 questionnaire in NHS I (*n = *86,672, age range 42–67 y) and 1989 questionnaire in NHS II (*n = *116,677, age range 25–42 y) served as the baseline population for our analyses, because the information of rotating night shift work was first available in these years. Participants were excluded if they had diabetes, heart diseases, stroke, or cancer at baseline (*n = *14,766 in NHS I, and *n = *7,222 in NHS II), missing information on diabetes diagnosis date (*n = *1,452 in NHS I, and *n = *749 in NHS II), or age (*n = *234 in NHS II), or shift work measures (*n = *1,185 in NHS I, and *n = *557 in NHS II). Finally, 69,269 women in NHS I and 107,915 women in NHS II were included in our analyses.

### Ascertainment of Rotating Night Shift Work

In NHS I, participants were asked at a single time point in 1988 how many years in total they had worked rotating night shifts (defined as at least three nights/month in addition to days and evenings in that month), with eight prespecified response categories: never, 1–2, 3–5, 6–9, 10–14, 15–19, 20–29, and ≥30 y. In NHS II, participants were asked the same question in 1989, with seven prespecified response categories: never, 1–2, 3–5, 6–9, 10–14, 15–19, and ≥20 y. The information was updated in 1991, 1993, 1997, 2001, and 2005 in NHS II. The 1991, 1993, and 1997 questionnaires collected information about the total number of months during which the nurse had worked rotating night shifts in the past 2 y with prespecified response categories: none, 1–4 mo, 5–9, 10–14, 15–19, and ≥20 mo. Additionally, in 2001, gaps were filled by asking for duration of rotating night shifts in 1993–1995, 1995–1997, 1997–1999, and 1999–2001. In 2005, data on the duration of rotating night shifts in 2001–2003 and 2003–2005 were collected. We assigned and added together midpoint values in years (or months) and calculated the total years of rotating night shifts for the women. In the final analysis, the participants were classified into five categories: never, 1–2, 3–9, 10–19, and ≥20 y of rotating night shift work.

### Ascertainment of Incident Type 2 Diabetes

In both cohorts, a supplementary questionnaire regarding symptoms, diagnostic tests, and hypoglycemic therapy was mailed to participants who reported a diagnosis of diabetes. A case of type 2 diabetes was considered confirmed if at least one of the following was reported on the supplementary questionnaire according to the National Diabetes Data Group criteria [11]: (1) one or more classic symptoms (excessive thirst, polyuria or frequent urination, weight loss, hunger) plus fasting plasma glucose levels of at least 7.8 mmol/l or random plasma glucose levels of at least 11.1 mmol/l; (2) at least two elevated plasma glucose concentrations on different occasions (fasting levels of at least 7.8 mmol/l, random plasma glucose levels of at least 11.1 mmol/l, and/or concentrations of at least 11.1 mmol/l after ≥2 h shown by oral glucose tolerance testing) in the absence of symptoms; or (3) treatment with hypoglycemic medication (insulin or oral hypoglycemic agent). The diagnostic criteria changed in June 1998, and a fasting plasma glucose of 7.0 mmol/l was considered the threshold for the diagnosis of diabetes instead of 7.8 mmol/l according to the American Diabetes Association criteria [12].

The self-reported type 2 diabetes diagnosis through supplemental questionnaire confirmation has been demonstrated to be highly accurate in a validation study: of 62 type 2 diabetes cases in NHS I who were confirmed by the supplementary questionnaire, 61 (98%) were reconfirmed by medical records [13]. Moreover, in another substudy to assess the prevalence of undiagnosed diabetes in NHS I, fasting plasma glucose and plasma fructosamine were measured in a random sample of participants (*n = *200) who did not report a previous diagnosis of diabetes. Only one (0.5%) of the women had an elevated fasting plasma glucose or plasma fructosamine level in the diabetic range, and her levels were barely above the diagnostic cutoffs [14]. By confirming all self-reported cases of diabetes, we exclude false-positive results, and the NHS I results suggest that the false-negative rate is low because most nurses have ready access to medical care, and only cases confirmed by the supplemental questionnaires were included.

### Assessment of Covariates

In the biennial follow-up questionnaires, we inquired and updated information on risk factors for chronic diseases, such as body weight, cigarette smoking, physical activity, family history of diabetes, menopausal status, and hormone use. Dietary information (including alcohol) was assessed using a validated semi-quantitative food frequency questionnaire every 4 y starting from 1986 (NHS I) and 1991 (NHS II) [15]. A low-risk diet score was defined as a diet low in trans-fat and glycemic load, while high in cereal fiber and ratio of polyunsaturated to saturated fat. The dietary score is a sum of the quintile values of the four nutrients/components with five representing the lowest-risk quintile in each dietary factor. This method was described in detail elsewhere [10]. Information on daily hours of sleep and snoring frequency was collected in 1986, 2000, and 2002 in NHS I, information about the participants' own education and her husband's education level was inquired in 1992 for NHS I.

### Statistical Analysis

Person-years for each participant were calculated from the return date of the baseline questionnaire to the date of diagnosis of type 2 diabetes, death, or the end of the follow-up period (June 30, 2008 for NHS I and June 30, 2007 for NHS II), whichever came first. Time-dependent Cox proportional hazards models were used to estimate the hazard ratios (HRs) of developing type 2 diabetes in rotating night shift workers. The comparison group was women who did not report a history of rotating night shift work. In the multivariate analysis, we adjusted for age (continuous), questionnaire cycle (each 2-y interval), ethnicity (white, nonwhite), family history of diabetes (yes, no), smoking status (never, past, current 1–14/d, 15–24/d, ≥25/d), alcohol intake (0, 0.1–4.9, 5.0–14.9, ≥15 g/d), physical activity (<3, 3–8.9, 9–17.9, 18–26.9, ≥27 MET-h/wk), current aspirin use (yes, no), menopausal status and hormone use (premenopausal, postmenopausal never users, postmenopausal past users, postmenopausal current users), oral contraceptive use (yes, no; NHS II participants only), and quintiles of total energy and dietary score. In additional analyses, we further adjusted for body mass index (BMI; <23, 23–24.9, 25–29.9, 30–34.9, ≥35 kg/m^2^) to examine the degree to which the association between type 2 diabetes and shift work was mediated by BMI. The above time-varying covariates included in the multivariate analyses were updated every 2 or 4 y, using the most recent data for each 2-y follow-up interval. The last value was carried forward for one 2-y cycle to replace missing values. If the last value was missing, then a missing value indicator was created. In a sensitivity analysis, we adjusted for waist circumference (which was assessed in 1986 for NHS I, and 1993 for NHS II) instead of BMI.

To further explore whether duration of shift work was associated with subsequent weight gain, we conducted a secondary analysis in NHS II because more detailed information was collected on duration of shift work and the participants were young and middle-aged women who were prone to weight gain. In this analysis, women with missing information on body weight were further excluded (*n = *252). Obesity status in 2007 and weight gain from 1989 to 2007 were used as the outcomes, and duration of shift work was updated until 2007 and was used as the exposure. We performed logistic regression analyses to estimate the odds ratio of obesity (defined by BMI ≥30 kg/m^2^) and excessive weight gain (more than 5% of baseline body weight) by duration of shift work category. We used linear regression to estimate the mean BMI or weight-gain difference corresponding to each 5-y increase in shift work, adjusting for aforementioned covariates including baseline BMI.

Proportional hazards assumption was tested with a time-dependent variable by including an interaction term between duration of rotating night shift work and months to events (*p*>0.05 for all tests). Tests for linear trend were conducted by assigning the median value to each category and modeling this value as a continuous variable. We pooled estimates from the two cohorts using the fixed-effect models because they were similar in terms of population characteristics, study design, and sample size. We also conducted a sensitivity analysis of using random-effects models, and the results were not materially altered. All *p-*values were two-sided, and data were analyzed with SAS 9.1 (SAS Institute Inc).

## Results

Baseline characteristics of the cohorts are reported in Table 1. The mean age at baseline was 53.9 (standard deviation [SD] 7.1) y in NHS I, and 34.3 (SD 4.7) y in NHS II. Of all women in NHS I, 59.0% reported ever having engaged in ≥1 y of rotating night shift work, with 11.3% reporting shift work for ≥10 y. In the younger cohort (NHS II) at baseline (1989), 61.9% reported ever having engaged in ≥1 y of shift work, with only 4.4% reporting shift work for ≥10 y. In 2001, 12 y postbaseline for the NHS II cohort, the mean age was 46.1 (SD, 4.7) y, and the two proportions related to shift work increased to 69.0% and 7.9%, respectively. In both cohorts, women with more years spent in rotating night shift work were older, more likely to have a higher BMI, and to be current smokers. In NHS I, women with more years of rotating night shift work were more likely to be diagnosed with hypertension, to report regular snoring and <6 h sleep. No appreciable differences in dietary factors were observed across durations of shift work.

**Table 1:** Age and age-standardized baseline characteristics of the study population at baseline by category of years spent in rotating night shift work.

Characteristics	Duration of Rotating Night Shift Work				
	Never	1–2 y	3–9 y	10–19 y	≥20 y
NHS I (1988)					
*n* (%)	28,367 (41.0)	16,941 (24.5)	16,146 (23.3)	4,909 (7.1)	2,906 (4.2)
Age (y)	53.5 (7.1)	53.1 (7.0)	54.5 (7.1)	54.7 (7.1)	57.0 (6.4)
BMI (kg/m^2^)	25.1 (4.6)	25.0 (4.5)	25.5 (4.8)	26.3 (5.1)	26.6 (5.5)
Physical activity (MET-h/wk)	14.8 (21.2)	15.9 (22.0)	16.4 (22.8)	16.0 (20.8)	17.8 (27.6)
Alcohol (g/d)	6.3 (10.7)	6.6 (10.8)	6.4 (10.7)	5.9 (10.8)	5.2 (10.3)
Total energy (kcal/d)	1,749 (519)	1,778 (516)	1,788 (532)	1,794 (538)	1,783 (559)
Diabetes dietary score	12.0 (2.4)	12.1 (2.4)	12.0 (2.4)	11.8 (2.4)	11.7 (2.4)
Cereal fiber (g/d)	4.5 (3.1)	4.6 (3.2)	4.4 (2.9)	4.3 (3.0)	4.3 (3.5)
Glycemic load	98.7 (18.8)	98.9 (18.5)	98.5 (18.6)	98.6 (18.9)	99.2 (19.3)
Polyunsaturated to saturated fat ratio	0.6 (0.2)	0.6 (0.2)	0.6 (0.2)	0.5 (0.2)	0.5 (0.2)
Trans-fat (% of energy)	1.7 (0.5)	1.7 (0.5)	1.7 (0.5)	1.7 (0.5)	1.7 (0.6)
Fruit and vegetables (servings/d)	4.9 (2.0)	5.0 (2.0)	5.0 (2.0)	5.0 (2.1)	4.9 (2.1)
Coffee (cups/d)	2.4 (1.7)	2.4 (1.7)	2.5 (1.7)	2.7 (1.8)	2.7 (1.9)
Red meat (servings/d)	1.2 (0.6)	1.2 (0.6)	1.2 (0.6)	1.3 (0.6)	1.2 (0.6)
Soft drinks (servings/d)	0.8 (1.0)	0.8 (1.0)	0.8 (1.1)	0.9 (1.2)	0.9 (1.2)
Race, white	98	98	98	97	97
Current smoker	17	17	20	25	24
Premenopausal	39	39	38	37	36
Current hormone therapy user	21	22	21	19	17
Regular aspirin user	67	69	67	67	66
Family history of diabetes	27	28	30	31	32
Hypertension	24	23	26	28	29
High cholesterol	22	22	22	22	22
Short sleep duration, ≤6 h/d	23	24	27	33	35
Regular snoring	7	7	8	9	10
NHS II (1989)					
*n* (%)	41,084 (38.1)	31,471 (29.2)	30,546 (28.3)	4,673 (4.3)	141 (0.1)
Age (y)	34.3 (4.7)	34.1 (4.8)	34.1 (4.6)	36.7 (3.4)	40.4 (2.5)
BMI (kg/m^2^)	23.8 (4.8)	23.7 (4.7)	24.3 (5.2)	25.3 (5.9)	24.8 (5.6)
Physical activity (MET-h/wk)	22.9 (34.2)	25.2 (36.4)	27.3 (40.0)	28.2 (41.7)	25.1 (54.9)
Alcohol (g/d)	3.0 (6.0)	3.2 (6.2)	3.3 (6.1)	3.0 (6.4)	1.4 (4.5)
Total energy (kcal/d)	1,769 (538)	1,792 (546)	1,803 (556)	1,812 (573)	1,803 (497)
Diabetes dietary score	12.0 (2.7)	12.1 (2.7)	12.0 (2.7)	11.8 (2.7)	11.4 (2.4)
Cereal fiber (g/d)	6.2 (2.5)	6.2 (2.5)	6.0 (2.4)	5.8 (2.4)	5.5 (2.2)
Glycemic load	124.3 (18.9)	124.0 (18.7)	123.6 (18.8)	123.7 (19.0)	122.1 (18.5)
Polyunsaturated to saturated fat ratio	0.54 (0.14)	0.54 (0.14)	0.53 (0.14)	0.53 (0.14)	0.50 (0.14)
Trans-fat (% of energy)	1.6 (0.5)	1.6 (0.5)	1.6 (0.5)	1.6 (0.5)	1.6 (0.6)
Whole grain (g/d)	23.4 (13.9)	23.4 (13.7)	23.0 (13.5)	21.8 (13.0)	21.4 (12.5)
Fruit and vegetables (servings/d)	5.0 (2.5)	5.2 (2.6)	5.3 (2.7)	5.3 (2.8)	5.8 (2.9)
Coffee (cups/d)	1.5 (1.5)	1.6 (1.5)	1.6 (1.5)	1.6 (1.6)	1.6 (1.7)
Red meat (servings/d)	1.0 (0.6)	1.0 (0.6)	1.0 (0.6)	1.0 (0.6)	1.1 (0.6)
Soft drinks (servings/d)	1.3 (1.2)	1.4 (1.3)	1.5 (1.3)	1.6 (1.4)	1.6 (1.5)
Race, white	96	95	95	95	71^a^
Current smoker	12	13	15	19	26
Premenopausal	97	97	97	96	96
Current hormone therapy user	3	4	4	5	3
Current oral contraceptive user	13	13	13	11	14
Regular aspirin user	11	11	11	14	7
Family history of diabetes	32	33	34	36	37
Hypertension	5	5	5	7	5
High cholesterol	10	10	10	11	7

Data were expressed as mean (SD) or percentage, unless otherwise specified. The number of missing data in NHS I: BMI (*n = *4,825); physical activity level (*n = *126); diet information (*n = *14,612); smoking status (*n = *145); menopausal status and hormone use (*n = *3,229); sleep duration (*n = *8,964); snoring frequency (*n = *9,031). The number of missing data in NHS II: BMI (*n = *252); physical activity level (*n = *393); diet information (*n = *19,496); smoking status (*n = *473); menopausal status and hormone use (*n = *649).

a Because of small sample size in this category, the percentage might not be accurate. In 2001 for NHS II, the corresponding percentage was 95%.

A total of 6,165 incident type 2 diabetes cases were documented during 1,260,694 person-years in NHS I, and 3,961 cases during 1,865,320 person-years in NHS II. In the age- and questionnaire-cycle–adjusted models, duration of rotating night shift work was monotonically associated with an increased risk of type 2 diabetes in both cohorts (*p* for trend <0.001) (Table 2). Compared with women who reported no rotating night shift work, the HRs (95% CIs) for participants with 1–2, 3–9, 10–19, and ≥20 y of rotating night shift work were 0.99 (0.93–1.06), 1.17 (1.10–1.25), 1.42 (1.29–1.55), and 1.64 (1.46–1.83) in NHS I, as well as 1.13 (1.04–1.23), 1.34 (1.23–1.45), 1.76 (1.57–1.96), and 2.50 (2.00–3.14) in NHS II, respectively. This association was slightly attenuated after controlling for other covariates except BMI. Additional adjustment for updated BMI further attenuated the association, although it was still monotonic and significant, and the corresponding HRs (95% CI) were 1.00 (0.94–1.07), 1.06 (0.99–1.13), 1.09 (0.99–1.20), and 1.20 (1.07–1.34) in NHS I, as well as 1.07 (0.98–1.16), 1.05 (0.97–1.14), 1.10 (1.00–1.25), and 1.44 (1.15–1.80) in NHS II, respectively. The pooled HRs (95% CI) were 1.03 (0.98–1.08), 1.06 (1.01–1.11), 1.10 (1.02–1.18), and 1.24 (1.13–1.37, *p* for trend <0.001).

**Table 2:** Hazard ratio of type 2 diabetes by years of working rotating night shifts.

Studies	Duration of Rotating Night Shift Work					*p-*Value for Trend	Hazard Ratio per 5 y of Shift Work
	Never	1–2 y	3–9 y	10–19 y	≥20 y		
**NHS I (1988–2008)**							
Cases/person-years	2,322/519,988	1,388/311,468	1,534/292,014	549/86,844	372/50,380	—	—
Incidence rate (per 1,000 person-years)	4.5	4.5	5.3	6.3	7.4	—	—
Age-adjusted model	1.00	0.99 (0.93–1.06)	1.17 (1.10–1.25)	1.42 (1.29–1.55)	1.64 (1.46–1.83)	<0.001	1.14 (1.12–1.17)
Multivariate-adjusted model 1	1.00	1.01 (0.95–1.08)	1.15 (1.08–1.23)	1.32 (1.20–1.45)	1.47 (1.32–1.64)	<0.001	1.11 (1.08–1.13)
Multivariate-adjusted model 2	1.00	1.00 (0.94–1.07)	1.06 (0.99–1.13)	1.09 (0.99–1.20)	1.20 (1.07–1.34)	<0.001	1.05 (1.02–1.07)
**NHS II (1989–2007)**							
Cases/person-years	1,000/584,808	1,053/540,270	1,377/599,813	449/128,835	82/11,593	—	—
Incidence rate (per 1,000 person-years)	1.7	2.0	2.3	3.5	7.1	—	—
Age-adjusted model	1.00	1.13 (1.04–1.23)	1.34 (1.23–1.45)	1.76 (1.57–1.96)	2.50 (2.00–3.14)	<0.001	1.23 (1.19–1.27)
Multivariate-adjusted model 1	1.00	1.12 (1.02–1.22)	1.28 (1.18–1.39)	1.54 (1.38–1.73)	2.13 (1.70–2.67)	<0.001	1.18 (1.14–1.22)
Multivariate-adjusted model 2	1.00	1.07 (0.98–1.16)	1.05 (0.97–1.14)	1.11 (1.00–1.25)	1.44 (1.15–1.80)	0.026	1.05 (1.01–1.08)
**Pooled results** a							
Age-adjusted model	1.00	1.04 (0.99–1.10)	1.24 (1.18–1.30)	1.55 (1.45–1.66)	1.78 (1.61–1.96)	<0.001	1.17 (1.15–1.20)
Multivariate-adjusted model 1	1.00	1.05 (1.00–1.11)	1.20 (1.14–1.26)	1.40 (1.30–1.51)	1.58 (1.43–1.74)	<0.001	1.13 (1.11–1.14)
Multivariate-adjusted model 2	1.00	1.03 (0.98–1.08)	1.06 (1.00–1.11)	1.10 (1.02–1.18)	1.24 (1.13–1.37)	<0.001	1.05 (1.04–1.06)

Multivariate-adjusted model 1: adjusted for age (continuous), alcohol consumption (0, 0.1–4.9, 5.0–14.9, ≥15 g/d), physical activity level (<3, 3–8.9, 9–17.9, 18–26.9, ≥27 MET-h/wk), smoking status (never, past, current 1–14/d, current 15–24/d, current ≥25/d), race (white, nonwhite), menopausal status and hormone use (premenopausal, postmenopausal never users, postmenopausal past users, postmenopausal current users), oral contraceptive use (yes, no; in NHS II), family history of diabetes (yes, no), current aspirin use (yes, no), quintiles of total calorie, diabetes dietary score. Multivariate-adjusted model 2: model 1 plus updated BMI category (<23, 23–24.9, 25–29.9, 30–34.9, ≥35 kg/m^2^).

a The results were pooled using fixed-effect models.

In NHS I, further adjustment for sleep duration, snoring frequency, and the nurse's own and her husband's education levels did not change the results, and no significant interaction between rotating night shift work and sleep duration was found. In both cohorts, further adjustment for baseline histories of hypertension and hypercholesterolemia did not appreciably change the results, and the pooled HRs (95% CI) were 1.02 (0.97–1.08), 1.05 (0.99–1.10), 1.09 (1.02–1.17), and 1.23 (1.11–1.36). The results were the same in white participants only and no interaction with ethnicity was found. In a sensitivity analysis of adjustment for waist circumference instead of BMI, the HRs (95% CI) were 1.01 (0.95–1.08), 1.13 (1.06–1.20), 1.24 (1.13–1.36), and 1.36 (1.21–1.51) in NHS I, as well as 1.11 (1.02–1.21), 1.23 (1.13–1.33), 1.44 (1.28–1.61), and 1.83 (1.46–2.30) in NHS II, respectively. The results did not appreciably change in sensitivity analyses of adjustment for waist-hip ratio or continuous BMI instead of categorical BMI.

In the model without BMI, every 5-y increase of rotating night shift work was associated with an 11% (95% CI 8%–13%) and 18% (95% CI 14%–22%) elevated risk of type 2 diabetes in NHS I and II, respectively, and this estimate was reduced to 5% (95% CI 2%–7%) and 5% (95% CI 1%–8%) after adjustment for BMI, respectively. No significant interaction between rotating night shift work and baseline BMI was found.

**Figure 1:**
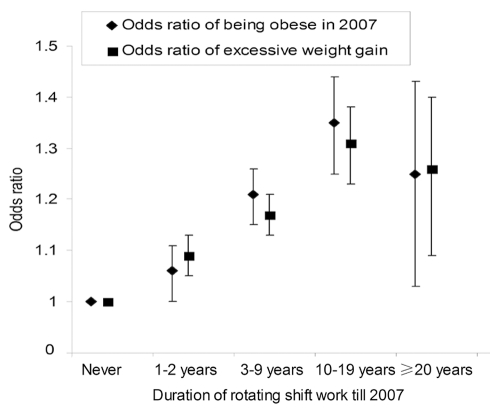
Rotating night shift work and risk of obesity and weight gain in Nurses' Health Study II. The figure shows the odds ratio (95% CI) of being obese in 2007 and excessive weight gain between 1989 and 2007 by categories of rotating night shift work in Nurses' Health Study II. Excessive weight gain was defined as weight gain between 1989 and 2007 of more than 5% of the baseline body weight in 1989. The model was adjusted for baseline age, BMI, alcohol consumption, physical activity level, smoking status, ethnicity, menopausal status and hormone use, oral contraceptive use, family history of diabetes, current aspirin use, quintiles of total calorie and dietary score in 1989.

In a secondary analysis conducted in NHS II (*n = *107,663), we found that rotating night shift work was associated with an elevated risk of obesity and excessive weight gain during the follow-up period (Figure 1). In the multivariate analysis, each 5-y increase in rotating night shift work was associated with an increase of 0.17 units in BMI (95% CI 0.14–0.19) and an increase of 0.45 kg in weight gain (95% CI 0.38–0.53). In addition, women who started their shift works between 1989 and 2007 were also at a high risk of weight gain: the corresponding increase was 0.39 (95% CI 0.28–0.50) units in BMI and 1.02 (95% CI 0.70–1.33) kg in weight gain for each 5-y increase in rotating night shift work, when we excluded those reporting a history of shift work before 1989.

## Discussion

In the two prospective cohort studies with 18–20 y of follow-up, we found that women had a modestly increased risk of type 2 diabetes after extended periods of rotating night shift work. This association appears to be partly mediated through body weight. The NHS I and II cohorts captured different age groups, but the results were fairly consistent across cohorts suggesting that the increased risk of type 2 diabetes was not limited to a particular age group.

Two prospective studies in male Japanese workers revealed that alternation/shift work was an independent risk factor for impaired glucose metabolism and diabetes [7],[8]. These studies, however, did not have information on duration of rotating night shift work. In a previous analysis using NHS II data with 6 y of follow-up (1993–1999), we found that the positive association between years in rotating night shift work and diabetes was entirely mediated by BMI [10]. In that analysis, a history of rotating night shift work ≥10 y was associated with a 64% increased risk of type 2 diabetes in the age-adjusted model. The association disappeared after adjusting for BMI. There were multiple limitations to that analysis including a small sample size (356 total type 2 diabetes cases, 35 cases in women with ≥10 y of shift work), a short follow-up duration (6 y), and few women with longer years of shift work (≥20 y). In our updated analysis, we found that BMI mediated only part of the association, and women with ≥20 y of shift work still had a 44% increased risk of developing type 2 diabetes after adjusting for BMI in NHS II. Our current analysis provides compelling evidence that an extended period of rotating night shift work is associated with a moderately increased risk of type 2 diabetes, which was not completely explained by BMI.

The increased risk of type 2 diabetes associated with rotating night shift work is also consistent with previously reported positive associations of rotating shift work with obesity and/or weight gain [2], metabolic syndrome [3],[4], and cardiovascular disease [16]. There are several potential mechanisms underlying this association. First, a wide range of biological processes are regulated by the circadian rhythms, including sleep-wake cycles, body temperature, energy metabolism, cell cycle, and hormone secretion. Rotating night shift work is generally associated with chronic misalignment between the endogenous circadian timing system and the behavior cycles. This circadian misalignment has been found to result in adverse metabolic and cardiovascular consequences, including a decrease in leptin, an increase in glucose and insulin, an increase in mean arterial blood pressure, and reduced sleep efficiency [17]. Furthermore, the increase in glucose seems to be the result of an exaggerated postprandial glucose response [17].

Second, unfavorable changes in health behaviors (such as increased smoking and irregular meals) related to rotating night shift workers may partly explain the observed association. However, our study and previous studies showed only small differences in nutritional intake and eating patterns between daytime and shift workers [18]–[20], although shifting the time of food intake may influence the postprandial glucose and insulin levels [21],[22], and increase body mass [23]. Nevertheless, the long-term effects of eating habits on health status in rotating night shift workers remain unclear. In our study, women with more years of rotating night shift work were more likely to be current smokers, while no significant difference was found for total energy intake and dietary score between daytime and rotating night shift workers. Third, working in a rotational shift, particularly when it involves night work, may influence both quality and quantity of sleep [24]. Accumulating evidence from prospective studies suggests an increased risk of type 2 diabetes associated with sleep deprivation and sleep disorders [25],[26]. In our analysis, women with more years of rotating night shift work were more likely to sleep ≤6 h per day and snore regularly in NHS I. However, the association between rotating night shift work and type 2 diabetes was not explained by sleep duration and snoring frequency in NHS I. Other factors such as disturbed socio-temporal patterns (resulting from atypical work hours leading to family problems, reduced social support, and stress) and unfavorable changes to biomarkers (e.g., cholesterol and other lipids, plasminogen, blood pressure, and cardiac activity) might also impact the association [16],[27].

The strengths of this study include its prospective design, the large sample size and detailed information on a wide range of potential confounders, and long-term follow-up. To the best of our knowledge, this is the largest cohort study in a female population investigating the association between rotating night shift work and diabetes. We are also aware of several limitations of this study. First, our study populations primarily consisted of white female nurses. The homogeneity of our study participants minimized confounding by socioeconomic status and enhanced the response rate and the quality of the questionnaire data, but the generalizability of our data to other populations, particularly men and other racial or ethnic groups, may be limited. The two above-mentioned studies in Japanese male workers, however, also suggested a positive association between rotating night shift work and diabetes compared with day workers [7],[8]. Second, information on rotating night shift work was self-reported, which may have led to misclassifications of the exposure. However, because of the prospective nature of our study, these misclassifications were more likely to have attenuated our results towards the null.

Third, the underascertainment and misclassification of diabetes outcome are possible because the incident cases were self-reported. However, our validity studies indicated that self-reported diabetes was highly reliable in this group of health professionals. The association could be biased if there was differential detection of diabetes by categories of work schedules. However, when analyses were restricted to symptomatic cases of type 2 diabetes, findings were not materially altered, suggesting that surveillance bias by work schedules was unlikely.

Furthermore, although we controlled for a wide range of covariates including lifestyle factors (e.g., smoking, physical activity, and diet quality), the possibility of unmeasured and residual confounding cannot be fully excluded. Lower socioeconomic status and less healthy lifestyles have been associated with shift work [28]. However, controlling for the participant's own and her husband's education levels and working status in a sensitivity analysis in NHS I did not alter the results. On the other hand, health-related selection could also lead to an underestimate of the association [29],[30]. It is possible that some rotating night shift workers who remained on night shift schedules were healthier than those who worked on daytime schedules or switched back to day routines or withdrew from work for health reasons [29],[30] and thus were less likely to accumulate a longer duration of rotating night shift work. Therefore, the association between duration of rotating night shift work and diabetes may be underestimated if the reference group included some of the women who worked on daytime schedules or withdrew from work for health reasons. Lastly, since our study is observational in nature, causality could not be inferred. Randomized clinical trials may better address the issue of causality, but the study design may not be feasible for this case.

In conclusion, the results from these two large, well-established, long-term cohort studies suggest a positive association between rotating night shift work and diabetes risk. Long duration of shift work was also associated with greater weight gain. Additional studies are needed to confirm our findings in men and other ethnic groups and to further investigate the underlying mechanisms for the association. Because a large proportion of the working population is involved in some kind of permanent night and rotating night shift work, our study has potential public health significance. Recognizing that rotating night shift workers are at a higher risk of type 2 diabetes should prompt additional research into preventive strategies in this group.

## Abbreviations

BMI: body mass index
NHS: Nurses' Health Study
SD: standard deviation
